# Association between small intestinal bacterial overgrowth and deep vein thrombosis

**DOI:** 10.1093/gastro/gow004

**Published:** 2016-04-04

**Authors:** Andre Fialho, Andrea Fialho, Aldo Schenone, Prashanthi Thota, Arthur McCullough, Bo Shen

**Affiliations:** ^1^Department of Internal Medicine, The Cleveland Clinic Foundation, Cleveland, OH, USA and; ^2^Department of Gastroenterology and Hepatology, The Cleveland Clinic Foundation, Cleveland, OH, USA

**Keywords:** small intestine bacterial overgrowth, deep vein thrombosis, risk factors

## Abstract

**Objective:** Small intestinal bacterial overgrowth (SIBO) has been associated with several diseases. The association between SIBO and deep vein thrombosis (DVT) has not been investigated. This study was aimed to investigate the frequency and risk factors for the development of DVT in patients tested for SIBO.

**Methods:** All 321 eligible patients were included from the Cleveland Clinic Gastrointestinal Motility Lab databank from January 2008 to January 2014. Patients who were evaluated with glucose hydrogen/methane breath test as well as Doppler ultrasonography for suspected DVT were included. Patients with catheter-related DVT were excluded. The primary outcomes were the frequency and risk factors (including SIBO) for DVT in this patient population.

**Results:** Of the 321-case cohort, 144 patients (44.9%) tested positive for SIBO, and 53 (16.5%) had ultrasonographic findings of DVT. SIBO evaluation *before* the evaluation of DVT occurred in 201 patients (median time from the breath test to ultrasonography: 27 months; interquartile range [IQR]: 11.0–45.0 months), and SIBO evaluation *after* evaluation for DVT occurred in 120 patients (median time from ultrasonography to the breath test: 30 months; IQR: 11.8–54.3 months). In the univariate analysis, DVT was associated with family history of thromboembolic events (35.8% *vs* 16.0%, *P*=0.001), chronic kidney diseases (CKD; 26.4% *vs* 13.4%, *P*=0.019) and the presence of SIBO (69.8% *vs* 39.9%, *P*<0.001). In the multivariate analysis, family history of thromboembolic events (odds ratio [OR]: 3.39; 95% confidence interval [CI]: 1.67–6.87; *P*<0.001), CKD (OR: 2.23; 95%CI: 1.04–4.74; *P* = 0.037), and the presence of SIBO (OR: 3.27; 95% CI: 1.70–6.32; *P* < 0.001) remained independently associated with DVT.

**Conclusion:** SIBO was found to be associated with DVT. The nature of this association warrants further investigation.

## Introduction

Small intestinal bacterial overgrowth (SIBO), defined as excessive colonization of the proximal small bowel by gram-negative aerobic and anaerobic bacteria, has been associated with nonalcoholic steatohepatitis (NASH) [1], Parkinson’s disease [2], inflammatory bowel disease (IBD) [3] and irritable bowel syndrome [4].

Venous thromboembolism (VTE) is characterized by clot formation in the venous system and manifests commonly by deep vein thrombosis (DVT) and pulmonary embolism (PE). The classic Virchow’s triad of damage to the vessel wall, venous stasis and hypercoagulability is implicated in its pathogenesis [5]. DVT occurs most often in the calf or thighs but can also originate in the proximal veins and is the primary cause of pulmonary thromboembolism [6]. Several diseases such as IBD and chronic kidney disease (CKD) have been implicated in the development of DVT, mainly by changes in coagulation factors and the production of pro-inflammatory mediators [7–9].

Lipopolysaccharide (LPS), produced by gram-negative bacteria, can induce a variety of systemic inflammatory responses. Bacterial LPS produced during sepsis has been implicated in changes in protein C anticoagulant pathway with an increased risk of thrombosis, especially in the microvasculature [10]. LPS has also been shown to cause thrombosis in large vessels in animal models [11]. There are no published studies in literature evaluating a possible role of quantitative changes in intestinal bacteria such as SIBO and the development of DVT. The aim of this study was to determine the frequency and risk factors for DVT in the patient population tested for SIBO.

## Patients and Methods

Patients were identified from the databank of the Gastrointestinal Motility Lab at the Cleveland Clinic. A total of 326 consecutive eligible patients tested for SIBO using glucose hydrogen (H_2_)/methane (CH_4_) breath test from January 2008 to January 2014 and who also had venous ultrasound study for suspected DVT were evaluated in this retrospective case-control. Detailed demographic and clinical data pertaining to SIBO and DVT were obtained. The study was approved by our Institutional Review Board.

### Inclusion and exclusion criteria

Inclusion criteria were patients with (i) glucose H_2_/CH_4_ breath test for evaluation of SIBO and (ii) Doppler ultrasonography of the upper and/or lower extremities performed at our institution. Patients with superficial thrombophlebitis or catheter-induced deep venous thrombosis were excluded. Mesenteric vein or portal vein thrombosis were not evaluated in this study.

### Study and control groups

The study group consisted of patients with SIBO, and the control group were those without SIBO. These groups were further subdivided according to the presence and absence of DVT.

### Diagnostic criteria

The diagnosis of SIBO was made by glucose H_2_/CH_4_ breath test. Patients were submitted to standard protocol. The H_2_/CH_4_ breath concentration was expressed in parts per million (p.p.m.) and measured by gas chromatography in basal conditions and every fifteen minutes for at least 3 hours after the administration of an oral loading dose of glucose (50 g in 250 ml of sterile water). The test was considered positive for SIBO when ≥ 1 of the criteria were present: H_2_ and/or CH_4_ increase >20 p.p.m. above basal value or H_2_ and/or CH_4_ increase >12 p.p.m. between the minimum and maximum values after glucose ingestion [12].

The diagnosis of upper or lower extremity DVT was made using venous ultrasound in either outpatient or inpatient setting. Gray scale with compression maneuvers, color Doppler and spectral Doppler at rest and with augmentation were performed. Upper extremity DVT was defined as a clot in the ulnar, radial or interosseous veins, brachial vein, axillary vein or subclavian vein. Lower extremity DVT was defined as a clot in the anterior tibial, posterior tibial, or peroneal veins, popliteal vein, femoral or iliac veins. Family history of VTE in first-degree family members was evaluated. VTE was defined as DVT or PE in these individuals.

### Study variables and outcome measurements

A total of 17 variables were studied. Clinical variables included were age, sex, setting where the ultrasound was performed (outpatient clinic, emergency department or inpatient), smoking history, family history of VTE in first-degree relatives, past medical history of cancer, diagnosis of CKD, diagnosis of IBD, diagnosis of systemic lupus erythematosus (SLE), diagnosis of cirrhosis, associated gastrointestinal conditions, use of steroids and use of estrogen/oral contraceptive pills (OCP). History of genetic thrombophilia including factor V Leiden mutation, prothrombin gene mutation, protein C and S deficiency was also reviewed. The primary outcomes were to evaluate the frequency of DVT in patients with SIBO and the associated risk factors for DVT in this population.

## Statistical Analysis

Continuous variables were presented as mean ± standard deviation (SD) or N%. Univariate analysis was performed to identify potential variables associated with SIBO. Student *t* tests or non-parametric Wilcoxon rank sum tests were used for continuous factors, and Pearson chi-square tests were used for categorical variables. Multivariate logistic regression analysis was performed to assess the risk factors associated with DVT in SIBO patients. An automated stepwise variable selection method performed on 1000 bootstrap samples was used to choose the final multivariable model. A *P-*value < 0.05 was considered statistically significant. All statistical analyses were performed using SPSS software version 22 (SPSS Statistics for Windows, Version 22.0. Armonk, NY: IBM Corp. 2013.)

## Results

The medical records of 326 patients were reviewed. Of these, five had upper extremity DVT that was catheter induced and were excluded from the study. A total of 321 patients who fulfilled inclusion criteria were evaluated in this study. Patients were subdivided based on their breath test results into SIBO positive (*n* = 144, the study group) or SIBO negative (*n* = 177, the control group). Patients were further subdivided according to the presence (*n* = 53) or absence (*n* = 268) of DVT. In all of the 53 patients with DVT, thrombosis occurred in the lower extremities.

### Interval in months between SIBO and DVT evaluation

A total of 201 patients had SIBO evaluation *before* evaluation of DVT with a median interval of 27 months (IQR = 11.0–45.0 months). A total of 120 patients had SIBO evaluation *after* evaluation for DVT with a median interval of 30 months and IQR = 11.8–54.3 months).

### Frequency of SIBO in the total population and associated gastrointestinal conditions

Among the 321 patients included, 144 (44.9%) were diagnosed with SIBO. Of the 144 patients with SIBO, 74 (51.4%) had associated gastrointestinal conditions as follows: history of bariatric surgery (4.2%), *Clostridium difficile* infection (2.0%), gastroparesis (3.5%), celiac disease (0.7%), cholecystitis (0.7%), diverticulosis (1.4%), gastroesophageal reflux (24.3%), irritable bowel disease (2.0%), hepatitis C (0.7%), intestinal obstruction (1.4%), pancreatitis (4.2%), peptic ulcer disease (4.9%), sphincter of Oddi dysfunction type II (0.7%) and rectal abscess (0.7%) (Table 1).

**Table 1.** Associated gastrointestinal conditions in patients with small intestinal bacterial overgrowth

Variables	SIBO positive (*n* = 144)
Bariatric surgery	6 (4.2%)
*Clostridium difficile* colitis	3 (2.0%)
Gastroparesis	5 (3.5%)
Celiac disease	1 (0.7%)
Cholecystitis	1 (0.7%)
Documented diverticulosis	2 (1.4%)
Gastroesophageal reflux	35 (24.3%)
Irritable bowel syndrome	3 (2.0%)
Hepatitis C	1 (0.7%)
Intestinal obstruction	2 (1.4%)
Pancreatitis	6 (4.2%)
Peptic ulcer disease	7 (4.9%)
Sphincter of Oddi dysfunction type II	1 (0.7%)
Rectal abscess	1 (0.7%)

### Frequency of DVT in the total population and in patients with SIBO

A total of 53 (16.5%) of the 321 patients included in this study were found to have imaging consistent with DVT. In all of the 53 patients, DVT occurred in the lower extremities. Among the 144 patients with SIBO, 37 (25.7%) had concomitant diagnosis of DVT compared with 16 (9.0%) of the 177 patients without SIBO (*p < *0.001). The presence of genetic thrombophilia occurred in 5 of 321 (1.6%) patients, with factor V Leiden mutation being the only diagnosis found.

### Comparison of demographic and clinical variables in patients with and without SIBO

There was no difference between the SIBO-positive and SIBO-negative patients with regard to age (65.1 ± 1.1 years *vs* 62.3 ± 1.4 years; *P* = 0.448), smoking (current 9.0% *vs* 6.2%; former 43.8% *vs* 43.5%; never 47.2% *vs* 50.3%; *P* = 0.488), IBD (9.7% *vs* 11.9%; *P* = 0.529), CKD (17.4% *vs* 14.1%; *P* = 0.443), ultrasound setting (outpatient appointment: 49.3% vs 50.8%; emergency department visit: 16.0% vs 10.7%; patients admitted to hospital: 34.7% vs 38.4%; p = 0.368) 49.3% vs 50.8%; 16.0 vs 10.7%; 34.7% vs 38.4%; P = 0.368) or location of DVT (external iliac vein 0.7% *vs* 1.1%; femoral vein 7.6% *vs* 2.8%; popliteal vein 11.8% *vs* 2.8%, tibial vein 6.9% *vs* 1.1 %; *P* = 0.346) as shown in Table 2. Patients who tested positive for SIBO were more likely to be males when compared with SIBO-negative patients (30.6% *vs* 19.2%; *P* = 0.013).

**Table 2.** Univariate analysis of the risk factors associated with small intestinal bacterial overgrowth

Variables	Total (*n* = 321)	SIBO + (*n* = 144)	SIBO – (*n* = 177)	*P*-value
Age, years	63.6 ± 0.8	65.1 ± 1.1	62.3 ± 1.4	0.448
Male sex, *n* (%)	78	44 (30.6)	34 (19.2)	0.026
Smoking, *n* (%)				0.488
Current	24	13 (9.0)	11 (6.2)	
Former	140	63 (43.8)	77 (43.5)	
Never	157	68 (47.2)	89 (50.3)	
Chronic kidney disease, *n* (%)	50	25 (17.4)	25 (14.1)	0.443
Inflammatory bowel disease, *n* (%)	35	14 (9.7)	21 (11.9)	0.529
Deep vein thrombosis, *n* (%)	53	37 (25.7)	16 (9.0)	<0.001
Ultrasound setting, *n* (%)				0.368
Outpatient	161	71 (49.3)	90 (50.8)	
Emergency room	42	23 (16.0)	19 (10.7)	
Inpatient	118	50 (34.7)	68 (38.4)	
DVT location, *n* (%)				0.346
External iliac vein	3	1 (0.7)	2 (1.1)	
Femoral vein	16	11 (7.6)	5 (2.8)	
Popliteal vein	22	17 (11.8)	5 (2.8)	
Tibial vein	12	10 (6.9)	2 (1.1)	

### Comparison of demographic and clinical variables in patients with and without DVT

Patients with DVT were found to have a higher family history of VTE in their first-degree relatives (35.8% *vs* 16.0%; *P* = 0.001), diagnosis of CKD (26.4% *vs* 13.4%; *P* = 0.019) and diagnosis of SIBO (69.8% *vs* 39.9%; *P* < 0.001). There was no difference among DVT-positive and DVT-negative patients regarding the setting of ultrasound test (outpatient, emergency room or inpatient), smoking history, cancer history, IBD, SLE, steroid use or estrogen/oral contraceptive use, hospital admission in the past 3 months, surgery in the past 3 months, malignancy in the past 3 months, infection in the past 3 months, > 48 hour hospitalization in the past 1 month, and current hospitalization in the past 3 months (Table 3).

**Table 3.** Univariate analysis of the risk factors associated with deep vein thrombosis

Variables	Total (*n* = 321)	DVT+ (*n* = 53)	DVT – (*n* = 268)	*P*-value
Age, years	63.6 ± 0.8	64.9 ± 2.2	63.3 ± 1.1	0.083
Male sex, *n* (%)	78	18 (34.0)	60 (22.4)	0.081
Ultrasound setting, *n* (%)				0.546
Outpatient	161	24 (45.3)	137 (51.1)	
Emergency room	42	6 (11.3)	36 (13.4)	
Inpatient	118	23 (43.4)	95 (35.4)	
Smoking, *n* (%)				0.416
Current	24	3 (5.7)	21 (7.8)	
Former	140	27 (50.9)	113 (42.2)	
Never	157	23 (43.4)	134 (50.0)	
Family history of venous thromboembolism, *n* (%)	62	19 (35.8)	43 (16.0)	0.001
Cancer history, *n* (%)	60	8 (15.1)	52 (19.4)	0.301
Chronic kidney disease, *n* (%)	50	14 (26.4)	36 (13.4)	0.019
Inflammatory bowel disease, *n* (%)	35	8 (15.1)	27(10.1)	0.332
Systemic lupus erythematosus, *n* (%)	17	4 (7.5)	13 (4.9)	0.498
Cirrhosis, n (%)	04	0 (0.0)	4 (1.5)	0.484
Small intestinal bacterial overgrowth, *n* (%)	144	37 (69.8)	107 (39.9)	<0.001
Corticosteroid use, *n* (%)	203	29 (54.7)	174 (64.9)	0.164
Estrogen/oral contraceptive use, *n* (%)	77	10 (18.9)	67 (25.0)	0.383
Hospital admission in the past 3 months, *n* (%)	167	22 (41.5)	145 (54.1)	0.189
Surgery in the past 3 months, *n* (%)	60	10 (18.9)	50 (18.7)	0.969
Malignancy in the past 3 months, *n* (%)	6	5 (9.4)	1 (0.4)	0.869
Infection in the past 3 months, *n* (%)	53	9 (17.0)	44 (16.4)	0.916
>48 hour immobilization in the past 1 month, *n* (%)	29	2 (3.8)	27 (10.1)	0.192
Current hospitalization, *n* (%)	113	21 (39.6)	92 (34.3)	0.721
Hospital admission in the past 3 months, *n* (%)	167	22 (41.5)	145 (54.1)	0.189
Surgery in the past 3 months, *n* (%)	60	10 (18.9)	50 (18.7)	0.969

### Multivariate analysis of variables in patients with and without DVT

In the stepwise multivariate logistic regression, family history of thromboembolic events (odds ratio [OR]: 3.39; 95% confidence interval [CI]: 1.67–6.87; *P*<0.001), CKD (OR: 2.23; 95% CI: 1.04–4.74; *P* = 0.037) and presence of SIBO (OR: 3.27; 95% CI: 1.70–6.32; *P* < 0.001) remained independently associated with DVT when evaluated in the model with cancer history, male sex, elderly age, SLE and IBD (Table 4 ).

**Table 4.** Stepwise multivariate analysis of risk factors associated with deep vein thrombosis

Variables	Adjustable odds ratio	95% confidence interval	*P*-value
Small intestinal bacterial overgrowth	3.27	1.70–6.32	<0.001
Chronic kidney disease	2.23	1.04–4.74	0.037
Family history of venous thromboembolism	3.39	1.67–6.87	<0.001
Male sex	1.84	0.92–3.68	0.086

Variables also included in the logistic analysis were cancer history, age, systemic lupus erythematosus and inflammatory bowel disease.

## Discussion

Small intestinal bacterial overgrowth and deep venous thrombosis are two separate disease entities that have a great impact in the health of affected patients with high rates of recurrence [13,14]. In the present study, SIBO was associated with DVT as an independent risk factor along with CKD and family history of thromboembolic events in first-degree relatives.

SIBO has been linked to several conditions such as NASH [1], Parkinson’s disease [2], IBD [3], irritable bowel syndrome [4], systemic sclerosis [15] and celiac disease [16]. Conditions that alter intestinal motility, such as scleroderma and diabetes, appear to predispose to SIBO [15,17]. SIBO may also be implicated in the development of NASH and Parkinson’s disease through increased production of pro-inflammatory markers [1,18].

DVT is characterized by deep venous blood clot formation. Conditions that promote stasis of the blood or increase hypercoagulability lead to acquired risk factors for DVT such as immobilization, trauma, surgery, antiphospholipid syndrome, hormone replacement therapy or use of oral contraceptives, pregnancy and puerperium. Deficiency of protein C, protein S and antithrombin and factor V Leiden mutation and prothrombin 20210A gene variant are genetic risk factors for DVT [19]. In addition, diseases such as IBD [20], CKD [8], SLE [21] and cancer [22] are commonly associated with an increased risk for clot formation.

Inflammatory states and higher levels of pro-inflammatory cytokines are known risk factors for DVT [23]. Furthermore conditions that increase bacterial translocation such as IBD have been implicated in the development of venous thrombosis through higher circulating levels of lipopolysaccharide (LPS) along with increased expression of Toll-like receptor 4 (TLR-4), an innate immune receptor responsible for LPS recognition [20]. TLR-4 is expressed by platelets and endothelial cells. LPS binding to TLR-4 causes pro-coagulatory activation [18] and may predispose to DVT.

In our study, SIBO was associated with DVT as an independent risk factor. We postulate that patients with SIBO are at a high risk for developing DVT due to a pro-inflammatory state caused by production of bacterial byproducts such as LPS, which may predispose to clot formation. Small intestinal bacterial overgrowth has been associated with increased release of interleukin (IL)-8 and enhanced expression of TLR-4 [24]. SIBO patients have also been shown to have high levels of mucosal IL-1a and b, which may be associated with gut inflammation and a pro-inflammatory state [25]. This association can also be bidirectional as patients with higher risk for DVT often have increased pro-inflammatory markers that may influence the gut immune system and alter gut microbiome, thereby promoting the development of SIBO.

Family history of venous thromboembolic events in first-degree relatives was found to be independently associated with SIBO in our study. This is in accordance with the literature as a familial history of venous thromboembolism has been shown to be a risk factor for venous thromboembolism such as DVT [26]. Chronic kidney disease, an established risk factor for venous thromboembolism, was also found in our study to be independently associated with DVT [8].

Known risk factors for DVT such as older age, the presence of IBD, cirrhosis, SLE, cancer history, use of corticosteroids or estrogen and smoking were not associated with DVT in our study. We postulate that this occurred due to the small sample size.

Our findings have several clinical implications. DVT occurred equally in outpatient and inpatient settings in our study. Patients with SIBO may benefit from a lower threshold for venous ultrasound evaluation even when presenting with subtle signs suggestive of DVT. Also, patients with DVT who have chronic gastrointestinal complaints may benefit from testing and treatment for SIBO. The treatment of one disease might benefit the other.

There are limitations to our study. First, the number of patients with DVT in this study was small, which may have compromised the power of the study. Second, there may have been selection and referral bias since the study was conducted at a tertiary center. That said, patients in our study may have had more severe disease and may not be representative of those seen in the general community. In addition, because this was a retrospective study, it was not possible to evaluate other risk factors for DVT such as immobility and genetic risk factors for DVT. It would be interesting to confirm a cause-and-effect association between SIBO and DVT by future prospective studies.

In conclusion, SIBO is associated with DVT as an independent risk factor. Patients with SIBO, therefore, may warrant closer monitoring and follow-up for findings suggestive of DVT. Further prospective studies are necessary to confirm SIBO as a risk factor for DVT and to establish if treatment for SIBO decreases the risk for DVT in this population.

*Conflict of interest statement*: none declared.
